# Effects of Hyperglycemia on Vascular Smooth Muscle Ca^2+^ Signaling

**DOI:** 10.1155/2017/3691349

**Published:** 2017-06-21

**Authors:** Nahed El-Najjar, Rashmi P. Kulkarni, Nancy Nader, Rawad Hodeify, Khaled Machaca

**Affiliations:** Department of Physiology and Biophysics, Weill Cornell Medicine Qatar, Doha, Qatar

## Abstract

Diabetes is a complex disease that is characterized with hyperglycemia, dyslipidemia, and insulin resistance. These pathologies are associated with significant cardiovascular implications that affect both the macro- and microvasculature. It is therefore important to understand the effects of various pathologies associated with diabetes on the vasculature. Here we directly test the effects of hyperglycemia on vascular smooth muscle (VSM) Ca^2+^ signaling in an isolated in vitro system using the A7r5 rat aortic cell line as a model. We find that prolonged exposure of A7r5 cells to hyperglycemia (weeks) is associated with changes to Ca^2+^ signaling, including most prominently an inhibition of the passive ER Ca^2+^ leak and the sarcoplasmic reticulum Ca^2+^-ATPase (SERCA). To translate these findings to the in vivo condition, we used primary VSM cells from normal and diabetic subjects and find that only the inhibition of the ER Ca^2+^ leaks replicates in cells from diabetic donors. These results show that prolonged hyperglycemia in isolation alters the Ca^2+^ signaling machinery in VSM cells. However, these alterations are not readily translatable to the whole organism situation where alterations to the Ca^2+^ signaling machinery are different.

## 1. Introduction

Diabetes is a complex multifactorial disease characterized by the onset of dyslipidemia, early hyperinsulinemia, and hyperinsulinemia, followed by pancreatic *β*-cell failure leading to hyperglycemia and insulin resistance [1–3]. This imbalance is associated with long-term complications and injury to multiple particularly susceptible organ systems, including the eye (retinopathy), kidney (nephropathy), peripheral nervous system (neuropathy), heart, and the vasculature (cardiovascular disease) [4]. There is significant evidence in the literature in both humans and animal models in support of the hypothesis that these pathologies are at least in part associated with the lack of glycemic control [4–6]. However, in addition to hyperglycemia, diabetes is associated with dyslipidemia, hyperinsulinemia, and increase reactive oxygen species [7]. This makes assignment of complications to a specific dysregulation at the whole organism level problematic.

Morbidity and mortality of individuals with diabetes result mainly from vascular dysfunction (VD) [3, 8–11]. Vascular complications associated with diabetes are divided into macrovascular complications, which include atherosclerosis, coronary artery disease, peripheral vascular disease, and microvascular complications such as retinopathy, nephropathy, and neuropathy [12–14]. Endothelial dysfunction plays an important role in vascular complications during diabetes [7]. Although in diabetes the mechanism of VD is complex and multifactorial involving multiple pathways [9, 13, 15, 16], chronic hyperglycemia is an important contributor to this process [3, 9, 10, 17].

Vascular smooth muscle (VSM) cells, existing in a differentiated quiescent state in the blood vessel wall, have a large repertoire of ion channels, receptors, signaling molecules, and contractile proteins essential for their contractile function [18, 19]. Because VSM contraction is dependent on a rise of cytoplasmic Ca^2+^ levels changes in VSM Ca^2+^ signaling have significant impact on determining vascular tone and peripheral resistance as both are dependent on resistance arteries diameter [20]. Consequently, any damage/modifications in the activity of key players involved in Ca^2+^ homeostasis are likely to be associated with VD. VSM cells play a key role in hyperglycemia-induced VD, including hypertension. Several lines of evidence suggest that oxidative stress caused by hyperglycemia provokes molecular pathologies that contribute to VD, leading to increased risk of adverse cardiovascular disease associated with diabetes [3, 10–12, 21–28]. Reactive oxygen species (ROS) are instrumental regulators of intracellular Ca^2+^ homeostasis and influence several other intracellular signaling pathways [29, 30]. Even though ROS generation is highly controlled in the vasculature, under physiological conditions, an increase in ROS generation under pathologic conditions contributes to vascular damage and cardiovascular disease [29]. Hyperglycemia-induced ROS is considered an important link between hyperglycemia and pathways of diabetic-related vascular complications [8, 16]. This is due to the fact that hyperglycemia-induced ROS is capable of damaging DNA and proteins and of inducing lipid peroxidation [23, 29]. This latter pathology affects ion transport across the cell membrane through two possible mechanisms: (1) inducing nonspecific leak of ions through the lipid bilayer and/or (2) modifying the physical properties of phospholipids in a way that alters the function of channels, pumps, and exchangers that are embedded within the lipid bilayer [29, 30]. For instance, inhibition of the sarcoplasmic/endoplasmic reticulum ATPase (SERCA) pump by hyperglycemia-induced ROS production results in an increase in intracellular Ca^2+^ [10]. This increase plays a role in the pathogenesis of vascular dysfunction by enhancing VSM cell migration [9, 10, 15]. The rat aortic cell line, A7r5, is a useful model for studying the effects of hyperglycemia on VSM function especially in the context of Ca^2+^ signaling since these cells have the complement of Ca^2+^ channels and pumps activities observed in freshly dispersed VSM cells [20, 31–33]. Searls et al. showed that in contrast to the defects observed in diabetic mice Ca^2+^ signaling pathways in A7r5 cells were not affected when the cells were shifted for short term from the physiological 5 mM glucose to the glycemic 25 mM and supraphysiological glucose 75 mM concentration was needed to see a significant effect [34].

Based on these findings our goal from the present work was to investigate the effect of prolonged exposure to high glucose levels on Ca^2+^ homeostasis in the A7r5 VSM cell line to better understand the potential pathology of hyperglycemia on the Ca^2+^ signaling machinery of VSM and potential implications on cardiovascular disease.

## 2. Materials and Methods

### 2.1. Materials

Dulbecco's modified Eagle's medium (DMEM), sodium pyruvate, penicillin/streptomycin, fetal bovine serum (FBS), phosphate-buffered saline (PBS), Trypsin/EDTA, N-acetyl-cysteine (NAC), mannitol, and phenylephrine (PE) were from Sigma Aldrich, St. Louis, MO, USA. Dimethylsulfoxide (DMSO) was obtained from Amresco (Amresco, USA). Thapsigargin (TG) was from Invitrogen and ionomycin was from Life Technologies.

### 2.2. Cell Culture

The embryonic rat aortic smooth muscle A7r5 cells (ATCC, Manassas, Virginia, USA) were grown, as recommended, in DMEM-high glucose (HG: 4.5 g/l equivalent to 25 mM), supplemented with 1% penicillin/streptomycin, 1% sodium pyruvate, and 10% FBS. To test the effect of glucose on the proliferation, metabolic activity, and Ca^2+^ signaling pathways in A7r5 cells, cells already cultured in HG (25 mM glucose) were shifted to DMEM-normal glucose (NG: 1 g/l equivalent to 5.5 mM) for more than 4 weeks. To rule out the osmotic effect induced by glucose, cells cultured in NG were shifted, for more than 4 weeks, to NG supplemented with mannitol (19.5 mM), a nonmetabolizable sugar. Cells were maintained at 37°C in a humidified atmosphere of 5% CO_2_ and 95% air. Throughout the manuscript, cells cultured under DMEM-HG, DMEM-NG, and DMEM-NG with mannitol are refereed to, respectively, as HG, NG, and OC streams. Primary human, aortic smooth muscle cells, from normal (NHVSMC) and diabetic (DHVSMC) individuals, were obtained from Lonza Walkersville (Walkersville, MD). Cells were grown, as recommended in smooth muscle basal medium supplemented with 5% FBS and a cocktail of different cytokines and growth factors obtained from Lonza Walkersville (Walkersville, MD). ReagentPack™ Subculture (kits including Trypsin/EDTA, Trypsin neutralizing solution, and HEPES buffered saline) designed specifically for the passaging of primary cell types was obtained from Lonza Walkersville (Walkersville, MD). Cells were maintained at 37°C in a humidified atmosphere of 5% CO_2_ and 95% air.

### 2.3. Cell Proliferation

The proliferation of A7r5 cells was determined using trypan blue dye exclusion counting using TC10 automated cell counter (Bio-Rad, CA, USA).

### 2.4. WST-1 Metabolic Activity Assay

To test whether the concentration of glucose affects the metabolic activity of the cells, A7r5 cells from HG, NG, and OC streams were plated, at equal density, in 96-well plates and shifted to HG or NG according to the followings: (NG:NG, NG: shifted to HG); (HG:HG, HG: shifted to NG), and (OC:OC, OC: shifted to HG). Three hours after plating the metabolic activity of the cells was evaluated by using the WST-1 (4-[3-(4-iodophenyl)-2-(4-nitrophenyl)-2H-5-tetrazolio]-1,3-benzenedisulfonate) assay (Roche Diagnostics GmbH, Mannheim, Germany). Using this assay, the ability of the cells to cleave by mitochondrial dehydrogenases the WST-1 tetrazolium salt to the red colored formazan allows assessing the metabolic activity of the cells under different glucose concentration. The absorbance was measured at 440 nm using Envision 2104 Multilabel Reader (Perkin Elmer, Massachusetts, USA).

### 2.5. Intracellular Calcium Measurements

A7r5 cells were cultured on 35 mm poly-d-lysine coated glass coverslips (MatTek corp, MA) and incubated in their respective media at 37°C. When 60–70% confluent, A7r5 cells were loaded with 2 *μ*M Fura-2AM (Invitrogen, NY, USA) for 30 min at 37°C. After incubation, cells were washed with PBS and incubated with Ca^2+^ containing Ringer buffer for 10 min at room temperature (RT) before analysis. The Ca^2+^ containing Ringer buffer contained (in mM) 120 NaCl, 5.0 KCl, 1.0 MgCl_2_, 2.0 CaCl_2_, 5.5 glucose, and 20 HEPES (pH 7.4). In Ca^2+^ free Ringer buffer, CaCl_2_ was replaced with equimolar MgCl_2_. For experiments that required low Na^+^, Ringer buffer NaCl was replaced with equimolar amount of N-methyl-D-glucamine (NMDG^+^). The imaging system included inverted epifluorescence microscope (Olympus IX71, PA) connected to a CoolSNAP HQ2 charged coupled device (CCD) camera. Image acquisition was performed using EasyRatioPro calcium imaging system (PTI, NJ).

Changes in cytosolic Ca^2+^ level were determined from the ratio of Fura-2AM fluorescence emission intensities following excitation at 340 and 380 nm.

### 2.6. Vascular Smooth Muscle Cells Contraction

Assessment of cell contraction was performed on A7r5 cells cultured on glass coverslips. Cells were washed with prewarmed Ca^2+^/Mg^2+^-free PBS and incubated in HBS for 10 min prior to live cell imaging. Cell contraction was visualized using an inverted microscope (Olympus, Japan) equipped with a LucPlan FLN 40x/0.60 objective. Images were acquired with a CCD camera (Olympus DP72, Germany) and processed using DP2-BSW software (Olympus Soft Imaging Solutions, Germany). Cell contraction was quantified by morphometric analysis using NIH software (ImageJ). Briefly, addition of 20 mM KCl induces the formation of contractile fibers that appears as protruded edges on the surface of contracted cells. To quantify contractile responses to KCl, contractile fibers were quantified using the edge detection function in ImageJ on thresholded time-lapse images.

### 2.7. Intracellular ROS Generation by DCFH

To test whether cells cultured under HG have higher reactive oxygen species (ROS) level than those cultured in NG and OC media, the level of ROS in cells cultured in media with different glucose concentration was examined using 2′,7′dichlorodihydrofluorescein diacetate (DCFH-DA) (Acros Organics, New Jersey, USA). In this assay, the DCFH-DA molecule passively diffuses into the cells and is cleaved and oxidized in the intracellular environment by ROS to the green fluorescence emitting compound, 2′,7′-dichlorofluorescein (DCF). Briefly, cells from HG, NG, and OC streams were plated, at equal density, in 96-well plates and after overnight starvation in 1% FBS, cells were incubated with 100 *μ*M DCFH-DA prepared in PBS at 37°C. 30 min after incubation, cells were shifted to HG, NG, or OC for an additional 30 min. Hydrogen peroxide (H_2_O_2_) (Sigma Aldrich, St. Louis, MO, USA) was used at 250 *μ*M as a positive control. DCF fluorescence was then determined, after cells lysis in 90% DMSO/10% PBS for 10 min in the dark, using a fluorescent plate reader (Envision 2104 Multilabel Reader, PerkinElmer, Massachusetts, USA) with 485 nm excitation and 520 nm emission wavelengths.

### 2.8. Western Blot Analysis

Total protein from HG, NG, and OC streams was extracted using RIPA buffer (Sigma Aldrich, St. Louis, MO, USA) supplemented with phosphatase and protease inhibitors. Protein extracts were quantified using Bradford method using Bio-Rad Protein Assay Dye Reagent (Bio-Rad, CA, USA) according to the manufacturer's protocol. Protein samples were mixed, respectively, with 10% and 25% of reducing agent and LDS buffer containing bromophenol blue for gel electrophoresis (Invitrogen, Carlsbad, CA, USA). An equal amount of protein lysate was subjected to gel electrophoresis on NuPAGE Bis-Tris or Tris-Acetate Gels (Invitrogen, Carlsbad, CA, USA) for 50 min at 200 V. Proteins were then transferred to a PVDF transfer membrane (Kisher Biotech, Germany) at 30 V for 30 min. After transfer, membranes were immunoblotted with the following primary antibodies: PMCA1, PMCA 4, and PMCA total (Affinity Bioreagents, Rockford, IL), SERCA 2 (Thermo Scientific, Pierce antibodies, Rockford, IL, USA), NCX1 (Swant, Bellinzona, Switzerland), STIM1 (Cell Signaling, Beverly, USA), Orai1 (Proteintech, USA), and IP3R1 (Millipore, USA). *β*-Actin, *α*-tubulin, and the secondary antibodies were from Sigma Aldrich (Sigma Aldrich, St. Louis, MO, USA). Following incubation with secondary antibodies, membranes were reacted with enhanced chemiluminescence western blot detection reagent (Amersham, GE Healthcare, UK). The luminescent reactivity was then measured using an image acquisition system, Gene SNAP, Geliance 600 Imaging system (Perkin Elmer, Massachusetts, USA). All membranes were stripped with Restore Plus Western Blot Stripping Buffer (Thermo Scientific, Rockford, IL, USA) and equal loading was then verified through reprobing the membranes with ß-actin/*α*-tubulin. Further densitometry analysis was performed using Gene Tools, Geliance 600 Imaging system (Perkin Elmer, Massachusetts, USA).

### 2.9. RNA Extraction and Real-Time PCR

RNA was extracted from A7r5 cells cultured in HG, NG, and OC using Qiagen RNAeasy extraction kit (Hilden, Germany) and reversed transcribed using High-Capacity cDNA reverse transcription kit (Applied Biosystems (AB)), all following the manufacturer's instructions. Quantitative real-time reverse transcriptase (qRT-PCR) was used to analyze the expression of the following genes (PMCA1, PMCA4, SERCA1, and SERCA2) using 2 *μ*L of template cDNA. *α*-Actin was used as housekeeping gene. Quantitect primers were obtained from Qiagen (Hilden, Germany). qRT-PCR was performed with the Fast SYBR Green Master Mix (2x) according to the manufacturer's instructions. Each PCR generated only the expected amplicon as shown by the negative first-deviation plots of the melting curve. Results were normalized to noninduced housekeeping gene-levels. Samples were analyzed in duplicate from three independent experiments.

### 2.10. Statistical Analysis

Results are presented as mean ± standard error of the mean (SE) from at least three independent experiments done each in triplicate. Comparison between the different groups was done using Nonparametric/Kruskal-Wallis test using IBM SPSS Statistics 23 software. The level of significance was set at 0.05.

## 3. Results

### 3.1. Long-Term Adaptation of A7r5 to Low or High Glucose Culture Conditions

A7r5 rat aortic smooth muscle cells are typically cultured in high glucose concentration of 25 mM. This concentration is hyperglycemic (HG) as the level of glucose in the blood of diabetic rats is around 17 mM, in contrast to the normoglycemic (NG) levels of 5.5 mM [34]. To begin to characterize the effect of hyperglycemia on Ca^2+^ signaling in VSM cells, we switched A7r5 cells initially cultured in HG to NG for up to 72 h and tested various Ca^2+^ signaling modalities, including basal Ca^2+^ levels, store-operated Ca^2+^ entry (SOCE), Ca^2+^ decay, and Ca^2+^ release but could not detect any alterations in Ca^2+^ signaling that correlate with the medium glucose concentrations (Supplemental Data, Figure  1, in Supplementary Material available online at https://doi.org/10.1155/2017/3691349). This data is in accordance with the study by Searls et al., who failed to show significant effect on Ca^2+^ when glucose levels were switched for a short time between 5 and 25 mM, prompting them to switch to 75 mM for short-term studies to replicate effect observed in diabetic mice [34].

Because complications associated with diabetes manifest themselves over prolonged periods of time (years in humans), we were interested in determining whether long-term exposure of VSM cells to HG concentrations affects Ca^2+^ signaling. We therefore incubated A7r5 cells for a minimum of 4 weeks in physiological glucose (5.5 mM) to mirror normoglycemia (NG) in the animal. Once cells were switched to the NG stream they were maintained under these culture conditions for the duration of the study. Alternatively another stream of A7r5 cells was cultured in 25 mM glucose representing the hyperglycemic group (HG). In addition, an osmotic control for the HG treatment was included (OC), where NG was supplemented with 19.5 mM mannitol, to separate effects due to hyperglycemia from those resulting from changes due to osmolarity at the high glucose concentrations.

**Figure 1:**
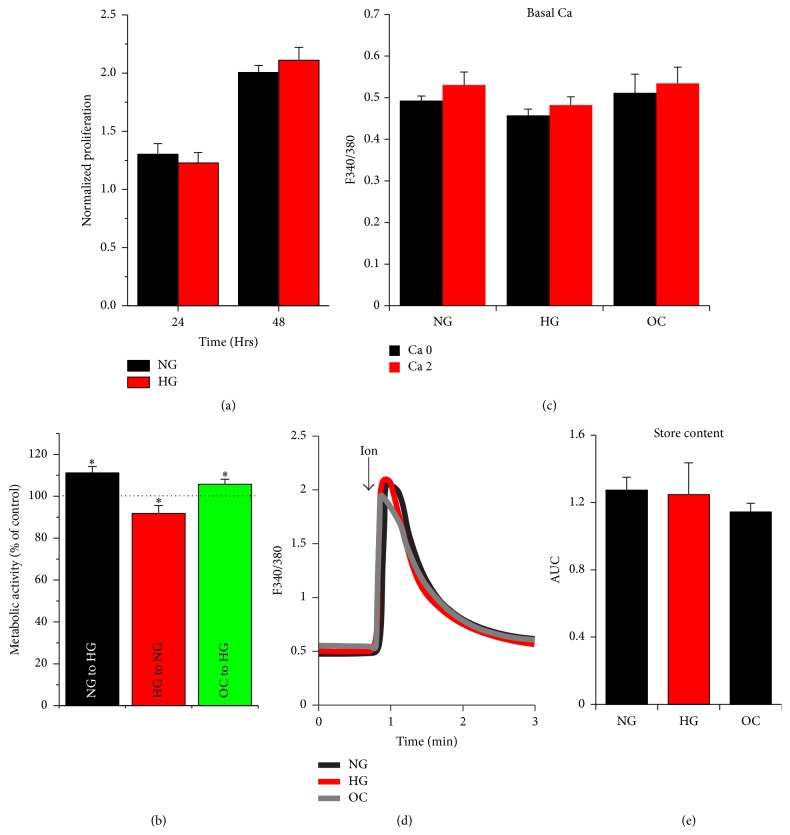
Effect of glucose on proliferation, basal Ca^2+^, and store Ca^2+^ content in A7r5 VSM cells. Proliferation (a), metabolic activity (b), basal Ca^2+^ level (c), and Ca^2+^ store content (d-e) were tested. Proliferation of A7r5 cells cultured under HG and NG was determined, 24 and 48 h after plating, using trypan blue dye exclusion counting (a). Metabolic activity of A7r5 cells from the different groups (HG, NG, and OC) shifted to HG or NG, measured 3 h later using the WST-1 assay (b). For basal Ca^2+^ levels and store content, cells cultured under HG, NG, or OC were loaded with Fura2-AM (2 *μ*M for 30 min) and the extent of Ca^2+^ release was determined following treatment of the cells with 2 *μ*M ionomycin (d-e). Data are presented as mean ± SE from at least three independent experiments done each in triplicate. ^*∗*^*p* < 0.05.

Surprisingly, long-term incubation of A7r5 cells in NG or HG does not significantly alter their proliferation rate (Figure 1(a)). This is likely due to the adaptation of the cells to the level of glucose used. In contrast, switching cells to different glucose concentrations results in small but detectable and statistically significant differences in their metabolic activity (Figure 1(b)). When A7r5 cells were shifted from NG or OC streams to HG for 3 h, this was associated with higher metabolic activity (*p* < 0.05) as compared to their respective controls. Similarly, when A7r5 cells were transferred from the HG stream to NG, they exhibited lower metabolic activity (*p* < 0.05). Furthermore, long-term incubation of A7r5 cells under the different streams NG, HG, or OC does not alter their basal resting cytoplasmic Ca^2+^ levels measured using Fura-2AM in Ca^2+^-free or Ca^2+^-containing extracellular solution (Figure 1(c)). We also measured intracellular store Ca^2+^ content in A7r5 cells cultured in the different streams using the ionomycin releasable Ca^2+^ pool in Ca^2+^-free media as illustrated in Figure 1(d). Ionomycin is a Ca^2+^ ionophore that is inserted into the lipid bilayer and nonspecifically equilibrates Ca^2+^. As such in Ca^2+^-free media, it releases Ca^2+^ trapped in intracellular stores such as the endoplasmic reticulum (ER). Incubating A7r5 cells in different glucose concentrations for extended time periods does not alter intracellular Ca^2+^ store content (Figures 1(d) and 1(e)).

**Figure 2:**
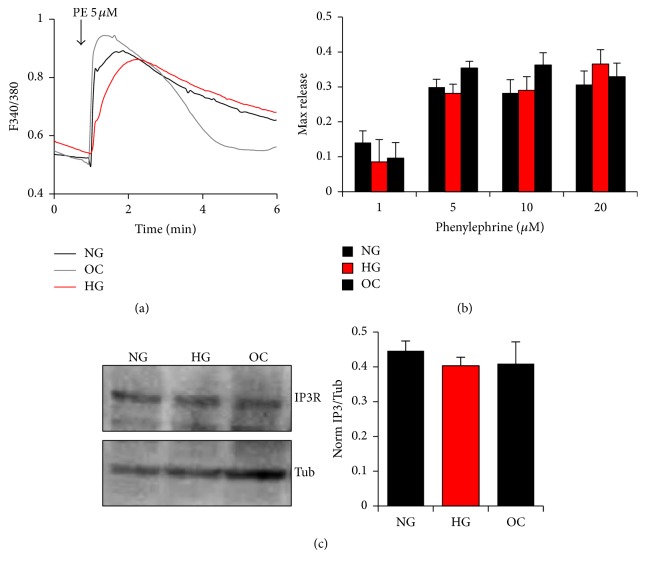
Effect of glucose normalization on Ca^2+^ release in A7r5 VSM cells. PE induced Ca^2+^ release (a, b) and IP3R expression (c) in response to different glycemic conditions. Cells cultured under HG, NG, or OC were loaded with Fura-2AM and Ca^2+^ transients imaged following exposure to different concentrations of PE (1–20 *μ*M) (a, b). IP3R expression from cells cultured under HG, NG, and OC was determined by western blot analysis and quantified, as described in Materials and Methods (c). Data are presented as mean ± SE from at least three independent experiments done each in triplicate.

Phenylephrine (PE) is an *α*-adrenergic receptor agonist that couples to trimeric G-proteins to activate phospholipase C (PLC) and generate inositol 1,4,5-triphosphate receptor (IP_3_). IP_3_ in turn binds to IP_3_ receptors (IP_3_Rs) on the ER membrane to gate them open and release intracellular Ca^2+^. The maximum levels of PE-induced Ca^2+^ release in cells cultured under the different glucose conditions were not statistically different arguing that the signal transduction cascade downstream of PE is not altered significantly when cells are cultured under hyperglycemia conditions (Figures 2(a) and 2(b)). Therefore, dramatic changes in the activity of the *α*-adrenergic receptor, G-proteins, or PLC are unlikely. Furthermore, protein levels of the IP_3_Rs are similar among the three tested streams (NG. HG, and OC) (Figure 2(c)), showing that glucose levels do not affect IP_3_Rs expression levels.

### 3.2. Store-Operated Ca^2+^ Entry (SOCE)

Agonist-mediated release of intracellular Ca^2+^ results in store depletion and the activation of a ubiquitous Ca^2+^ influx pathway known as store-operated Ca^2+^ entry (SOCE) [35]. This pathway is activated through the consorted action of two essential molecules: Orai1, a highly selective Ca^2+^ channel at the plasma membrane, and STIM1, a single pass ER membrane protein [36, 37]. The luminal domain of STIM1 has an EF-hand motif that senses Ca^2+^ store content [38, 39]. Ca^2+^ release in response to agonist stimulation lowers Ca^2+^ store content, which results in STIM1 oligomerization and its translocation to subplasma membrane cortical ER domains; where it recruits Orai1 and gates it open allowing Ca^2+^ influx [40–44]. Consequently, SOCE maintains Ca^2+^ homeostasis by ensuring the replenishment of the ER Ca^2+^ stores following store depletion.

**Figure 3:**
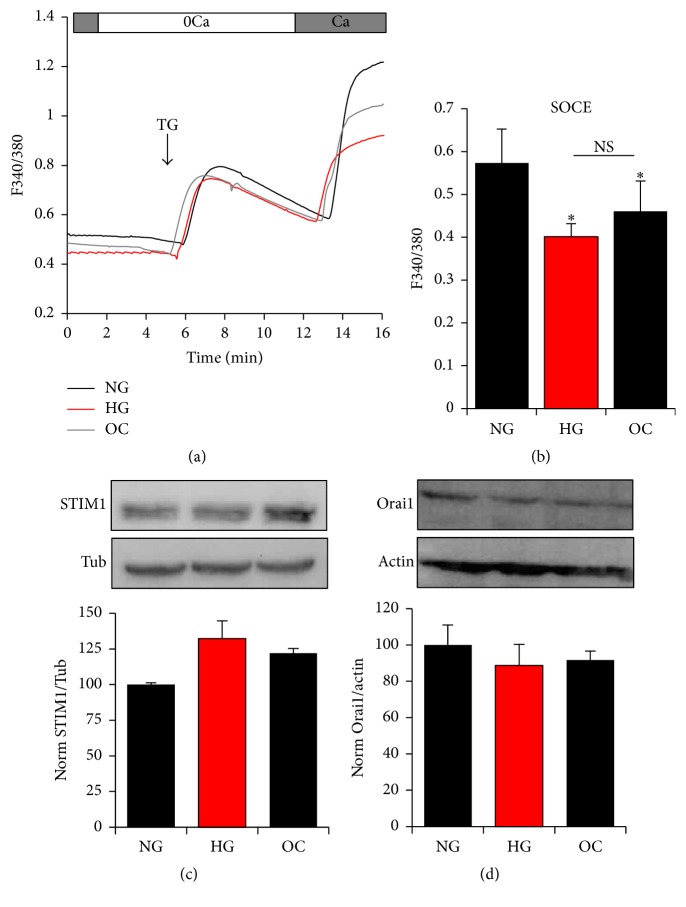
Effect of glucose Ca^2+^ influx in A7r5 VSM cells. Effect of HG and NG on store-operated Ca^2+^ entry (SOCE) (a, b) and on protein expression of STIM and Orai1 (c-d). Cells were loaded with Fura2-AM and SOCE stimulated after store depletion with 1 *μ*M thapsigargin (TG), an irreversible inhibitor of SERCA (a, b). Protein expression of STIM and Orai1 was determined between the different groups by western blot (c-d). Densitometry analysis was performed using Gene Tools, Geliance 600 Imaging system. Data are presented as mean ± SE from at least three independent experiments done each in triplicate. ^*∗*^*p* < 0.05; NS: not significant.

To evaluate the effect of glucose on SOCE in A7r5 cells, we measured SOCE levels using the classical Ca^2+^ readdition protocol after store depletion. For this assay Ca^2+^ stores were depleted with thapsigargin (TG), a specific nonreversible inhibitor of the sarcoplasmic reticulum Ca^2+^ ATPase (SERCA) that blocks store refilling and leads to store depletion due to an ill-defined continuous Ca^2+^ leak from the ER. Store depletion was performed in Ca^2+^-free conditions after which cells were switched to Ca^2+^-containing medium, which results in Ca^2+^ influx mediated by SOCE (Figure 3(a)). Incubation of A7r5 cells under hyperglycemic conditions results in a small but statistically significant decrease in SOCE (*p* < 0.034) (Figures 3(a) and 3(b)). A similar decrease in SOCE levels has been also observed in cells cultured under high osmolarity (OC) (*p* < 0.053), which mimic the osmotic conditions resulting from increased glucose levels in HG medium (Figure 3(b)). This argues that inhibition of SOCE under HG conditions is independent of glucose and likely due to the increased osmolarity.

We further tested the expression levels of the two primary SOCE proteins Orai1 and STIM1. Western blot analysis of A7r5 cells cultured under NG, HG, or OC shows that the expression levels of both STIM1 (Figure 3(c)) and Orai1 (Figure 3(d)) are not significantly different between the three groups. These results argue that extracellular glucose concentrations do not affect SOCE protein expression levels or SOCE activity.

### 3.3. Voltage Gated Ca^2+^ Channels (VGCC)

**Figure 4:**
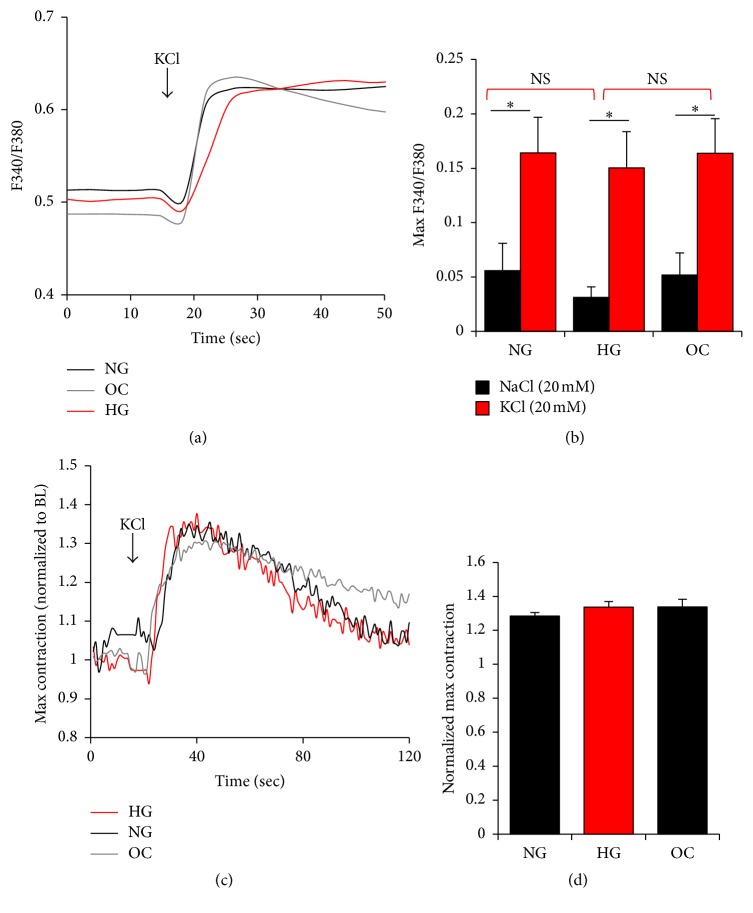
Effect of glucose on voltage gated channels (VGCC) in A7r5 VSM cells. Ca^2+^ transients (a, b) and VSM contraction (c, d) in response to a 20 mM KCl depolarizing pulse to activate VGCC in A7r5 cells. Cytosolic Ca^2+^ transients in Fura2-AM loaded A7r5 cells, cultured under HG, NG, and OC in response to 20 mM KCl (a, b). Effect of glucose normalization on A7r5 cells contractility in response to 20 mM KCl was determined as described in Materials and Methods. Max contraction is presented after normalization to basal level (c, d). Data are presented as mean ± SE from at least three independent experiments done each in triplicate. ^*∗*^*p* < 0.05; NS: not significant.

It is well established that Ca^2+^ entry through VGCCs, specifically the L-type Ca^2+^ channel, is important for VSM contractility and for regulating the myogenic response in resistance arteries [45–49]. Therefore, modulation of VGCC' activity could impact VSM contractility and function. In order to test the effects of extracellular glucose on VGCC we measured Ca^2+^ influx following a depolarization stimulus with KCl (Figure 4(a)). We could not observe any changes in the Ca^2+^ influx through VGCC, evaluated by measuring the peak amplitudes of KCl-induced Ca^2+^ transient (Figures 4(a) and 4(b)). As expected, the NaCl osmotic control did not stimulate Ca^2+^ influx as compared to KCl depolarization (Figure 4(b), black bars). To investigate whether glucose levels affect VSM contractility in response to Ca^2+^ influx through VGCC, we measured the contractility of A7r5 cells in response to KCl-induced depolarization (Figure 4(c)). As A7r5 cells are adherent, it is difficult to use cell shortening as a measure of contractility. However, contractile fibers, formed in cells stimulated with an agonist or with any Ca^2+^ mobilizing agent, are readily visible under light microscopy. Consequently, we used the previously described imaging approach that enables relative quantification of contractility [50]. Depolarization-induced contraction was similar among the three different groups (Figures 4(c) and 4(d)). These data are in agreement with the conclusion that glucose levels do not modulate the activity of VGCC or the resultant VSM contractions.

### 3.4. Ca^2+^ Leak from the Endoplasmic Reticulum (ER)

**Figure 5:**
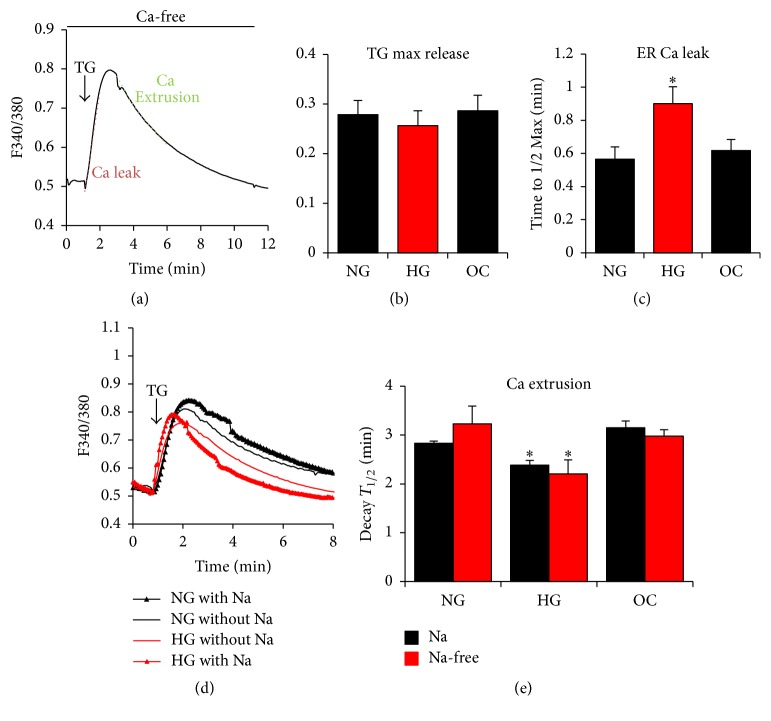
Effect of glucose on Ca^2+^ leak and Ca^2+^ extrusion in A7r5 VSM cells. A7r5 cells, cultured under HG, NG, and OC, were loaded with Fura2-AM and passive leak was monitored after store depletion with 1 *μ*M thapsigargin (TG), an irreversible SERCA inhibitor (a). Peak amplitude in response to TG (TG max release) and the time to maximum release are shown (b, c). Ca^2+^ extrusion, which is presumed to be due to the combined activity of PMCA and NCX, was monitored as the decay of the TG-induced Ca^2+^ transient in Ca^2+^ free conditions to avoid Ca^2+^ influx. To inhibit NCX, sodium was replaced with equimolar concentration of N-methyl-D-glucamine (d-e). Time to half (*T*_1/2_) decay was then measured and compared between the different conditions (d-e). Data are presented as mean ± SE from at least three independent experiments done each in triplicate. ^*∗*^*p* < 0.05.

Thapsigargin (TG) inhibition of the SERCA pump on the ER membrane demonstrates the presence of a passive leak pathway from the ER. The molecular mechanisms controlling this ER leak pathway remain controversial with roles proposed for the IP_3_Rs, presenilins, and the translocon machinery at the ER membrane as potential pathways [51–53]. In order to test whether the ER Ca^2+^ leak is altered in A7r5 cells cultured in HG, the maximum values and the time to half-max for the Ca^2+^ transient induced following treating cells with TG were measured in Ca^2+^-free Ringer buffer as illustrated in Figure 5(a). Under all three conditions NG, HG, and OC, the peak of the TG-induced Ca^2+^ transient was similar (Figure 5(b)), which is consistent with the ionomycin data (Figure 1(e)). These data show that Ca^2+^ store content is similar under all three conditions. In contrast, the time to reach half-max is significantly slower (*p* = 0.023) in cells grown under HG as compared to NG and OC (Figure 5(c)). Because the rise of the Ca^2+^ transient is partially dependent on Ca^2+^ leak from the ER, this argues that ER Ca^2+^ leak is inhibited when cells are cultured in HG.

### 3.5. Ca^2+^ Extrusion: Plasma Membrane Ca^2+^-ATPase (PMCA) and the Na^+^-Ca^2+^-Exchanger (NCX)

The plasma membrane Ca^2+^-ATPase (PMCA) and the sodium/calcium exchanger (NCX) are the primary mechanisms for Ca^2+^ extrusion out of the cell to maintain cytosolic Ca^2+^ homeostasis. To test whether Ca^2+^ extrusion is altered when cells are cultured under hyperglycemia condition, we used TG-dependent Ca^2+^ release in Ca^2+^-free conditions and calculated the rate of decay of the Ca^2+^ transient as a measure of Ca^2+^ extrusion out of the cell (Figure 5(a)). Under these conditions, Ca^2+^ influx is absent in Ca^2+^-free solution and Ca^2+^ reuptake into the ER is blocked by SERCA. Therefore, the only mechanisms that can lower cytoplasmic Ca^2+^ levels are the Ca^2+^ extrusion pathways. As such, the rate of decay of the Ca^2+^ transient under this experimental paradigm offers a measure of Ca^2+^ extrusion. To further differentiate between PMCA and NCX we performed these experiments under normal extracellular Na^+^ concentrations or with Na^+^ replaced with NMDG to inhibit the activity of NCX (Figures 5(d) and 5(e)). NCX uses the Na^+^ gradient across the cell membrane to extrude Ca^2+^ against its concentration gradient; therefore, in the absence of extracellular Na^+^, NCX activity is blocked. NCX in the absence of extracellular Na^+^ could run in reverse mode, thus transporting Ca^2+^ into the cell. However, this is unlikely to contribute under these experimental conditions since cells are incubated in Ca^2+^-free solutions.

**Figure 6:**
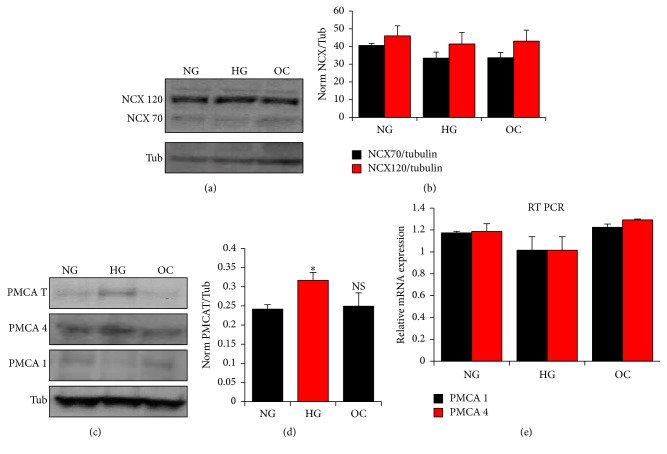
Effect of glucose on PMCA and NCX expression in A7r5 VSM cells. The protein expression of PMCA (total, isoform 1, and isoform 4) and NCX (full length: NCX 120 and proteolytic fragment: NCX 70) were compared in A7r5 cells cultured under HG, NG and OC by western blot analysis (a, c). Densitometry analysis was performed using Gene Tools, Geliance 600 Imaging system (b, d). Gene expression of PMCA 1 and 4 was monitored by quantitative RT-PCR (e). Data are presented as mean ± SE from at least three independent experiments done each in triplicate. ^*∗*^*p* < 0.05; NS: not significant.

The rate of Ca^2+^ extrusion in Na^+^-containing or NMDG solutions was similar for each glucose treatment (NG, HG) and for the osmotic control (OC), arguing that the primary Ca^2+^ extrusion pathway in A7r5 cells is PMCA (Figure 5(e)). However, incubating cells in HG for prolonged periods of time leads to a small but significant increase in PMCA activity (*p* = 0.027), revealed as a faster decay to half-max (*p* = 0.033) (Figure 5(e)). Consistent with the functional data, NCX protein expression was not statistically different between the different groups (Figures 6(a) and 6(b)), supporting the conclusion that the differential activity observed is due to enhanced PMCA activity. To determine if this increase is due to an increase in PMCA mRNA or protein expression, we focused on the two PMCA isoforms known to be expressed in VSM cells, PMCA1 and PMCA4 [54]. Western blot analyses show an increase in total PMCA protein levels in the HG cultures, that is due to PMCA4 (*p* = 0.04) but not PMCA1 increased protein expression levels (Figures 6(c) and 6(d); Supplemental Figure  2). In contrast, quantitative RT-PCR show no change in PMCA1 and PMCA4 mRNA levels between the different groups (Figure 6(e)).

### 3.6. Sarcoplasmic Reticulum Ca^2+^-ATPase (SERCA)

**Figure 7:**
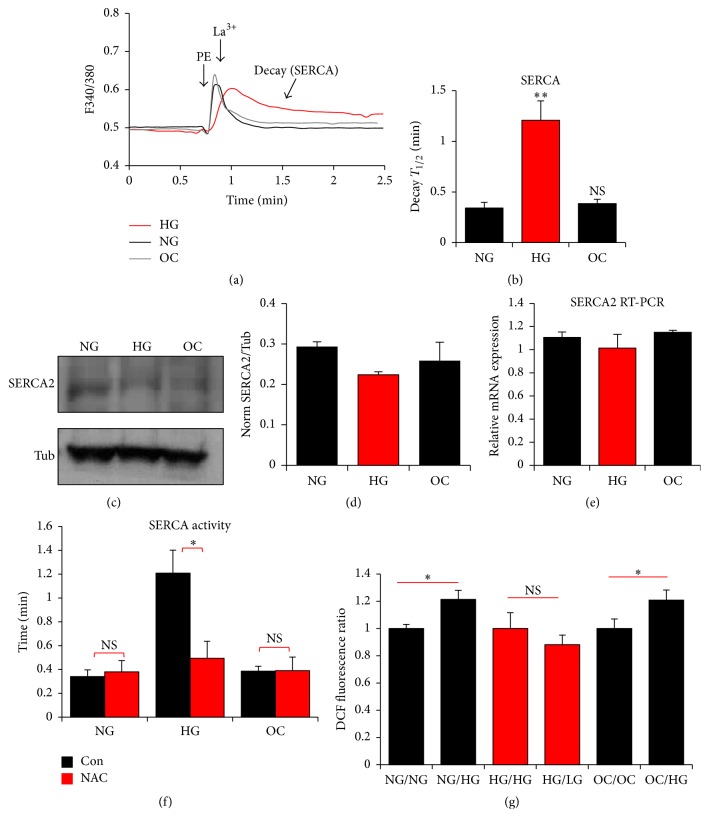
Effect of glucose on sarcoplasmic reticulum ATPase (SERCA) activity in A7r5 VSM cells. A7r5 cells, cultured under HG, NG, and OC, were loaded with Fura2-AM and cytosolic Ca^2+^ was monitored following treatment with 20 *μ*M PE and 1 mM lanthanum chloride (La^3+^) at the time points indicated by the respective arrows (a). Time to half decay after the La^3+^ treatment was then measured and compared between the different conditions (b). Protein and gene expression of SERCA from A7r5 cells cultured under the different condition were determined by western blot and real-time PCR, respectively (c–e). Effect of NAC on HG-induced SERCA compromised activity was determined in A7r5 cells cultured in the presence of NAC for 2 weeks prior to the determination of SERCA activity (f). The effect of HG on ROS generation in cells shifted for 30 min from NG and OC to HG and from HG to NG (g) was measured by the DCFH assay. Data are presented as mean ± SE from at least three independent experiments done each in triplicate. ^*∗∗*^*p* < 0.005.

To test for changes in the activity of the SERCA pump, which is responsible for Ca^2+^ store refilling after a Ca^2+^ transient, we released Ca^2+^ from intracellular store using phenylephrine and then at the peak of the Ca^2+^ transient a high concentration of lanthanum (La^3+^, 1 mM) was added to block both Ca^2+^ influx from the extracellular space and Ca^2+^ extrusion pathways [55]. Under these conditions where Ca^2+^ extrusion is blocked the decay kinetics of the Ca^2+^ signal back to baseline reflect SERCA activity (Figure 7(a)). Interestingly, the half-time of decay of the Ca^2+^ signal is significantly longer in cells incubated in HG conditions as compared to NG or OC conditions (*p* = 0.001) (Figures 7(a) and 7(b)). This shows that SERCA activity is inhibited when cells are cultured in HG conditions for extended time periods. Inhibition of SERCA activity is coupled to a small decrease in SERCA2 protein expression levels in HG culture conditions (Figures 7(c) and 7(d)). However, no changes in SERCA2 mRNA levels were detected (Figure 7(e)).

Several lines of evidence support the idea that hyperglycemia induces its damaging effect on SERCA through ROS generation. To validate this hypothesis in our system, A7r5 cells were cultured in HG, NG, or OC in the presence or absence of N-acetyl-cysteine (NAC), a universal radical scavenger, for 2 weeks.  Figure 7(f) shows that NAC significantly (^*∗∗*^*p* = 0.001) restores HG-induced SERCA compromised activity while no effect was seen on cells grown in NG or OC. These data confirm that oxidative stress is responsible for the decrease in SERCA activity of cells grown under HG. To further confirm the involvement of HG in ROS generation, intracellular ROS levels were determined using the ROS-sensitive fluorescence-generating probe DCF-DA assay. This assay shows that 30 min exposure to HG of cells already grown in NG or OC is sufficient to significantly (^*∗*^*p* < 0.05) increase ROS generation (Figure 7(g)).

### 3.7. Human Diabetic VSM Cells

To test whether the changes observed in the A7r5 model of cells cultured under hyperglycemic conditions apply to human VSM cells under diabetic conditions, we obtained primary aortic VSM cells from normal (NHVSMC) and diabetic (Type 2) individuals (DHVSMC) (Lonza, Walkersville, MD). Cells from normal and diabetic individuals were cultured in media containing NG levels (5.5 mM) supplemented with FBS and all necessary cytokines and growth factor as recommended by the supplier. Each batch of cells (normal and diabetic) was used within two weeks of culture. Cells were cultured in normal glucose levels to focus specifically on changes to the Ca^2+^ signaling machinery due to persistent irreversible damage as a result of diabetes and not to the culturing conditions. We used this approach based on the findings from the A7r5 model where alterations in Ca^2+^ signaling were observed only after prolonged exposure to hyperglycemia, strongly arguing that they are due to long lasting modifications.

**Figure 8:**
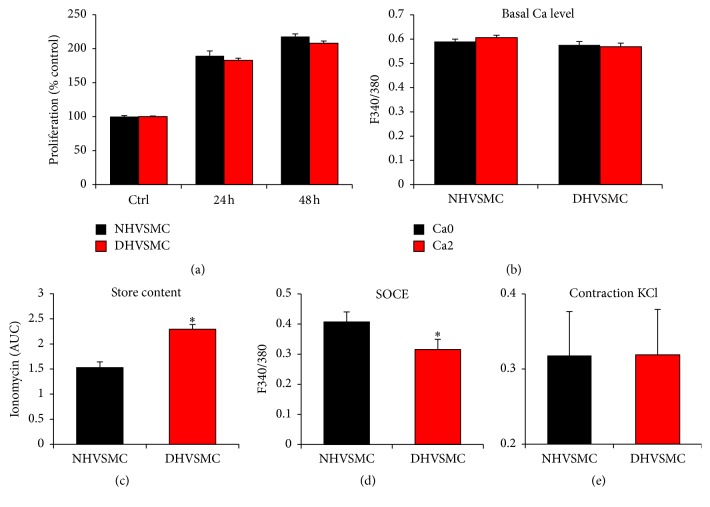
Proliferation and Ca^2+^ signaling in VSMC cells from normal (NHVSMC) and diabetic (DHVSMC) human aorta. For all the tests human normal and diabetic VSM cells were cultured under NG as recommended by the supplier. Cell proliferation was determined 24 and 48 h after plating, using the WST-1 assay (a). For Ca^2+^ basal levels and Ca^2+^ store content, cells were loaded with 2 *μ*M Fura-2AM for 30 min and the extent of Ca^2+^ release was determined following treatment of the cells with 2 *μ*M ionomycin (b-c). For SOCE measurement, cells were loaded with 2 *μ*M Fura-2AM for 30 min and SOCE was determined after store depletion with 1 *μ*M thapsigargin (TG) (d). Cell contractility in response to 20 mM KCl was determined as described in Materials and Methods (e). Data are presented as mean ± SE from at least three independent experiments done each in triplicate. ^*∗*^*p* < 0.05.

We applied the same approaches described above for A7r5 cells to test for changes in the Ca^2+^ signaling machinery in VSM cells from normal and diabetic individuals. No changes in proliferation rate (Figure 8(a)), basal Ca^2+^ levels (Figure 8(b)), or VSM contraction in response to a KCl depolarization (Figure 8(e)) were observed between cells from normal and diabetic individuals. In contrast, a significant increase (*p* = 0.009) in Ca^2+^ store content, based on the ionomycin releasable pool method, was detected in cells from diabetic individuals as compared to the normoglycemic control (Figure 8(c)). This increased store content was associated with a significant decrease (*p* = 0.044) in SOCE in diabetic VSM cells (Figure 8(d)). A similar decrease in SOCE was observed in A7r5 cells after prolonged exposure to high glucose concentrations, although this seems to be mostly attributable to an osmotic effect (Figure 3(b)).

**Figure 9:**
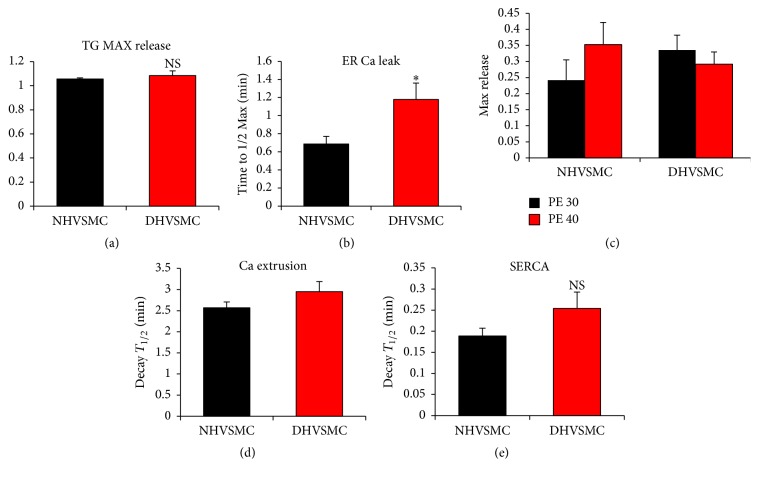
Ca^2+^ signaling pathways in normal (NHVSMC) and diabetic (DHVSMC) human aortic VSM cells. Cells were loaded with 2 *μ*M Fura-2AM for 30 min and passive leak was monitored after store depletion with 1 *μ*M thapsigargin (TG). Peak amplitude in response to TG (TG max release) and the time to half maximum release were compared between normal and diabetic cells (a, b). For the extrusion pathways the activity of PMCA and NCX was monitored by Ca^2+^ imaging after store depletion with 1 *μ*M TG. Time to half (*T*_1/2_) decay was then measured and compared between the different conditions (c). Cells were labeled with 2 *μ*M Fura-2AM for 30 min and Ca^2+^ release was imaged following treatment of the cells with 30 and 40 *μ*M of PE (d) or by using 30 *μ*M PE followed by 1 mM lanthanum chloride (La^3+^) (e). Time to half decay was then measured and compared between normal and diabetics cells (e). Data are presented as mean ± SE from at least three independent experiments done each in triplicate. ^*∗*^*p* < 0.05; NS: not significant.

Using the thapsigargin approach to assess ER Ca^2+^ leak, no changes in the maximal levels of Ca^2+^ release were detected (Figure 9(a)). This is in contrast to the enhanced ionomycin releasable Ca^2+^ pool in diabetic VSM cells (Figure 8(c)). This could be due to slow Ca^2+^ release from the ER following TG treatment, which is dependent on the rate of the passive ER Ca^2+^ leak. In contrast, ionomycin induces a rapid release from stores given its ionophore properties. The slow Ca^2+^ release in response to TG allows time for other pathways such as Ca^2+^ extrusion out of cell to act on the released Ca^2+^, which would mask the real store Ca^2+^ content.

Other aspects of Ca^2+^ signaling, including the response to the G-protein coupled agonist PE (Figure 9(c)), Ca^2+^ extrusion (Figure 9(d)), and importantly SERCA activity (Figure 9(e)), were not statistically different among the normal and diabetic VSM cells. Although SERCA activity showed a trend toward inhibition in diabetic human VSM cells, this did not reach statistical significance (Figure 9(e)). This is in contrast to the dramatic inhibition of SERCA activity observed in A7r5 cells exposed to high glucose for prolonged time periods (Figure 7(b)), which was the most pronounced alteration to Ca^2+^ signaling observed in this model.

To assess the expression levels of the different Ca^2+^ channels and transporters in human VSM cells, we used real-time PCR to evaluate mRNA levels of PMCA1, PMCA4, Orai1, Orai2, stim1, STIM2, and SERCA1-3. No significant changes were detected in any of the studied markers (Supplemental Figure  3).

## 4. Discussion

Ca^2+^ signaling is an integral signaling module involved in many aspects of cellular physiology. Specificity of Ca^2+^ signaling stems from the integrated contribution of different channels and transporters that move Ca^2+^ across disparate subcellular compartments leading to complex spatial and temporal dynamics to encode specific downstream cellular functions [56]. As such Ca^2+^ signals are able to encode various cellular responses based on the specific dynamics of Ca^2+^ transients. As such, alterations in the function or regulation of Ca^2+^ transporting proteins could have significant consequences on cellular and organismal physiology [56–58].

Several reports show that vascular dysfunction in the context of hyperglycemia is associated with alterations to multiple signaling pathways, including advanced glycation end-products (AGEs), PKC-DAG, and the hexosamine pathways [16, 24–28]. These pathways could directly or indirectly affect Ca^2+^ signaling, which in turn would contribute to vascular dysfunction (VD) by altering VSM or endothelial cell physiology. Interestingly, a recurring pathway that appears to be involved in the aforementioned mechanisms is the generation of reactive oxygen species (ROS) [24–28]. Furthermore, several lines of evidence suggest that oxidative stress caused by hyperglycemia increases the risk of the adverse cardiovascular events associated with diabetes [10, 11, 21, 26, 59]. For instance, in comparison to normal cells, an increase in ROS generation has been reported in smooth muscles from diabetic human uterine vessel [26]. It has been further shown that chronic treatment with antioxidants normalizes VD associated with diabetes supporting the deleterious role of ROS on vascular function [26, 59].

Our data show that short-term (up to 72 hrs) exposure of A7r5 vascular smooth muscle cells to hyperglycemic conditions does not result in any detectable changes in Ca^2+^ signaling. However, long-term culture of A7r5 cells in HG versus NG conditions leads to significant changes to Ca^2+^ signaling. Most significantly long-term exposure to hyperglycemic conditions is associated with an inhibition of both Ca^2+^ leak from the ER (Figure 5(c)) and SERCA-dependent Ca^2+^ reuptake into the ER (Figure 7(b)). The fact that these changes required culture under hyperglycemic conditions of more than 4 weeks argues that they are not driven by metabolic changes due to the increased glucose load, but rather that they are associated with modifications such as glycation or other cellular adaptations to the hyperglycemic conditions. Consistent with this conclusion, Fleischhacker et al. found no differential effect in the response of diabetic or normal smooth muscle cells to KCl and norepinephrine after they were isolated and cultured for 4 weeks [26]. Nevertheless, significant differences were observed when the cells were freshly isolated arguing that they are due to the pathophysiological state of the diabetic organism, potentially due to circulating factors, rather than irreversible changes at the cellular level. These effects were reversed following treatment with superoxide dismutase and when glycated products were removed. This argues that observed differential responses are not due to genetic modifications but require the presence of glycated products.

Furthermore, the contribution of HG-induced ROS to the observed SERCA inhibition has been previously reported [10]. Tong et al. showed that ROS-induced oxidation of Cys647 of SERCA inhibits nitric oxide- (NO-) induced-S-glutathionylation of SERCA leading to a decrease in its activity [10]. Therefore, to confirm that long-term normalization of glucose rescues SERCA activity by decreasing oxidative stress, we cultured A7r5 cells for 2 weeks in the presence of N-acetyl-cysteine (NAC), a strong radical scavenger. NAC normalized SERCA activity (Figure 7(f)). The involvement of ROS was further confirmed by the use of the DCFH assay that showed that exposure of cells already cultured in NG or OC media to HG for 30 min was sufficient to increase ROS levels (Figure 7(g)).

Culturing VSM cells under hyperglycemic conditions is an easy experimental approach to discern the effects of hyperglycemia on VSM function and has been used by several groups. The conclusion from our experiments and those of others are that there are long-term alterations that are associated with changes to VSM physiology exposed to hyperglycemia. We were as such interested in testing whether changes to Ca^2+^ signaling observed in A7r5 cells exposed for prolonged periods of time to hyperglycemia are replicated in primary VSM cells from human subjects with diabetes.

In diabetic human VSM cells, we observe an increase in ER Ca^2+^ store content (Figure 8(c)), inhibition of SOCE (Figure 8(d)), and an inhibition of passive Ca^2+^ lead from the ER (*p* = 0.02) (Figure 9(b)). Surprisingly, the most dramatic change to the Ca^2+^ signaling machinery observed in A7r5 cells, which is the inhibition of SERCA activity, was not statistically significant in human diabetic VSM cells (Figure 9(e)). This leaves the inhibition of ER Ca^2+^ leak as the only Ca^2+^ signaling pathway tested that consistently shows inhibition in both diabetic human VSM cells and the A7r5 cell culture model.

SOCE is inhibited in both the A7r5 model and human diabetic VSM cells; however, the fact that a similar inhibition was observed in the osmotic control in the A7r5 model in our hands casts doubt as to whether this inhibition is due to hyperglycemia per se.

There is compelling evidence from animal and clinical studies that some of the adverse effects of hyperglycemia on VD associated with diabetes are everlasting and cannot be reversed when glucose levels are improved or controlled [25]. In the context of Ca^2+^ signaling, we show here that the passive Ca^2+^ leak from the ER is consistently inhibited in VSM cells exposed to prolonged hyperglycemia both in humans and in culture model. In human diabetic VSM cells, consistently, this is associated with higher Ca^2+^ store content. However, these changes in Ca^2+^ dynamics were not associated with alteration to VSM Ca^2+^ signals in response to PE or to basal cytoplasmic Ca^2+^ levels. Therefore, their physiological significance remains unclear. Nonetheless, one could envision agonist stimulation in vivo leading to a more pronounced Ca^2+^ transient given the increased Ca^2+^ store content, which could result in a more sustained Ca^2+^ signal, thus affecting VSM physiology and contractility.

Hyperglycemia has also been shown to affect the microvasculature by impacting the activation state of the calcineurin-NFAT signaling cascade in smooth muscle cells of resistance arteries [60], a process that seems to be related to upregulation of SOCE in the endothelium in response to high glucose levels [37]. These changes could affect vascular health and contractility, thus leading to complications. There is indeed evidence supporting a decreased responsiveness of small mesenteric arteries to phenylephrine in diabetic as compared to control mice [61]. Therefore, hyperglycemia affects the responsiveness of microvessels and their contractility, which would ultimately contribute to the vascular dysfunction that is tightly associated with diabetes. Hence, understanding the molecular mechanisms through which hyperglycemia affects the function of both endothelial and VSM cells and their interactions, particularly in small resistance arteries, becomes critical.

Finally, an important take-home message from our experiments is that changes observed in a cell culture model of hyperglycemia need to be interpreted with caution as they are not readily translatable to VSM cells from diabetic subjects. This is not surprising given that the type and extent of exposure of VSM to hyperglycemia in the whole organism tend to be intermittent and it occurs in the context of a multitude of additional factors acting on VSM cells.

## Supplementary Material

Supplemental Figure 1: The effects of short term switch in hyperglycemia on Ca^2+^ signaling in VSM cells.
Supplemental Figure 2: Effect of glucose on protein expression of PMCA isoforms (1 and 4) in A7r5 cells cultured under HG, NG, and OC.
Supplemental Figure 3: Expression of Ca^2+^ channels and transporters in normal and diabetic VSM cells.
