# Supplementary material for: Effects of Hyperglycemia on Vascular Smooth Muscle Ca2+ Signaling
Source: Biomed Res Int. 2017 Jun 21;2017:3691349. doi: 10.1155/2017/3691349 (PMC5497615; doi:10.1155/2017/3691349)
Supplement: Supplementary file 1 — Supplemental Figure 1: The effects of short term switch in hyperglycemia on Ca2+ signaling in VSM cells. Supplemental Figure 2: Effect of glucose on protein expression of PMCA isoforms (1 and 4) in A7r5 cells cultured under HG, NG, and OC. Supplemental Figure 3: Expression of Ca2+ channels and transporters in normal and diabetic VSM cells. [file 3691349.f1.docx]

**Supplemental Data**

**Supplemental Figure 1:** **The effects of short term switch in hyperglycemia on Ca^2+^ signaling in VSM cells.** A7r5 cells already cultured in HG were shifted to NG for up to 72h. This short term change in glucose concentrations in the medium did not result in any significant changes in any of the following parameters: cellular basal levels (A), store operated calcium entry (SOCE) (B), decay (C), and in maximum calcium release after thapsigargin induced endoplasmic reticulum calcium depletion (D).

**Supplemental Figure 2:** **Effect of glucose on protein expression of PMCA isoforms (1 and 4) in A7r5 cells cultured under HG, NG, and OC.** Protein expression was compared among the different groups by western blot analysis. Densitometry analysis was performed and normalized to tubulin levels, used to correct for equal loading. Data are presented as mean ± SE from at least three independent experiments. * *P*<0.05. PMCA1 protein expression is not affected by glucose levels. Yet, a significant increase in the levels of PMCA 4 was observed when the cells are cultured under HG levels.

**Supplemental Figure 3:** **Expression of Ca^2+^ channels and transporters in normal and diabetic VSM cells.** Gene expression of RNA was extracted from NHVSM and DHVSM cells using Qiagen RNAeasy extraction kit (Hilden, Germany), and reversed transcribed using High-Capacity cDNA reverse transcription kit (Applied Biosystems (AB)), all following the manufacturer's instructions. Quantitative real-time reverse transcriptase (qRT-PCR) was used to analyze the expression of the following genes using 2μl of template cDNA: PMCA isoforms (1 and 4), Orai isoforms (1 and 2), Stim isoforms (1 and 2), SERCA isoforms (1, 2, and 3), and IP3R isoforms (1, 2, and 3) as indicated. α-actin was used as housekeeping gene. Quantitect primers were obtained from Qiagen (Hilden, Germany). qRT-PCR was performed with the Fast SYBR Green Master Mix (2x) according to the manufacturer's instructions. Each PCR generated only the expected amplicon as shown by the negative first-deviation plots of the melting curve. Results were normalized to non-induced housekeeping gene-levels. Samples were analyzed in duplicate from three independent experiments.

**Supplemental Figure 1**

**
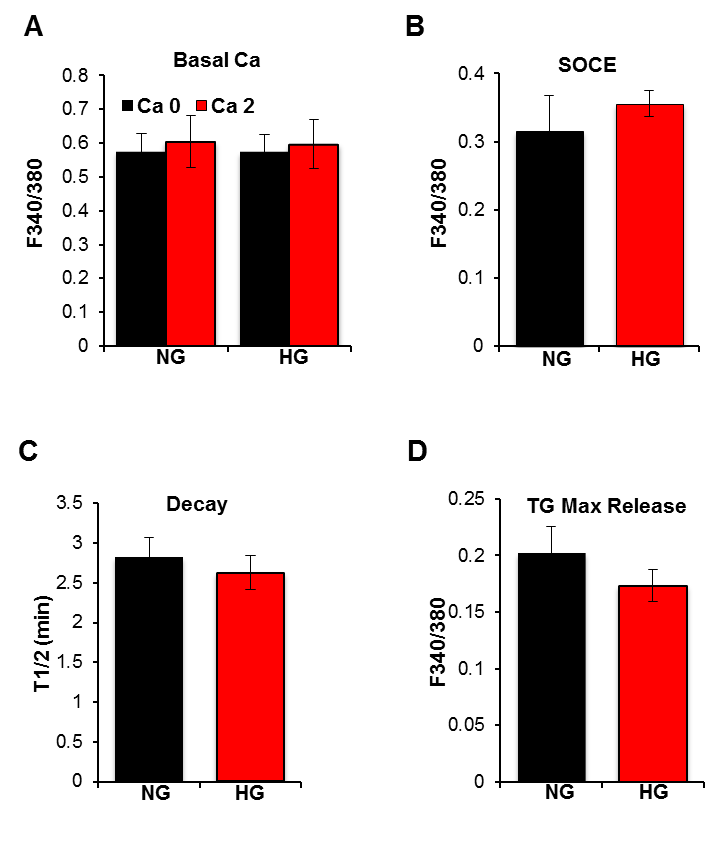
**

**Supplemental Figure 2**

**
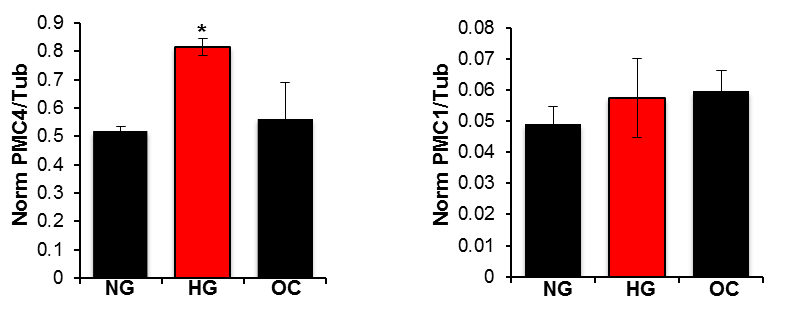
**

**Supplemental Figure 3**

**
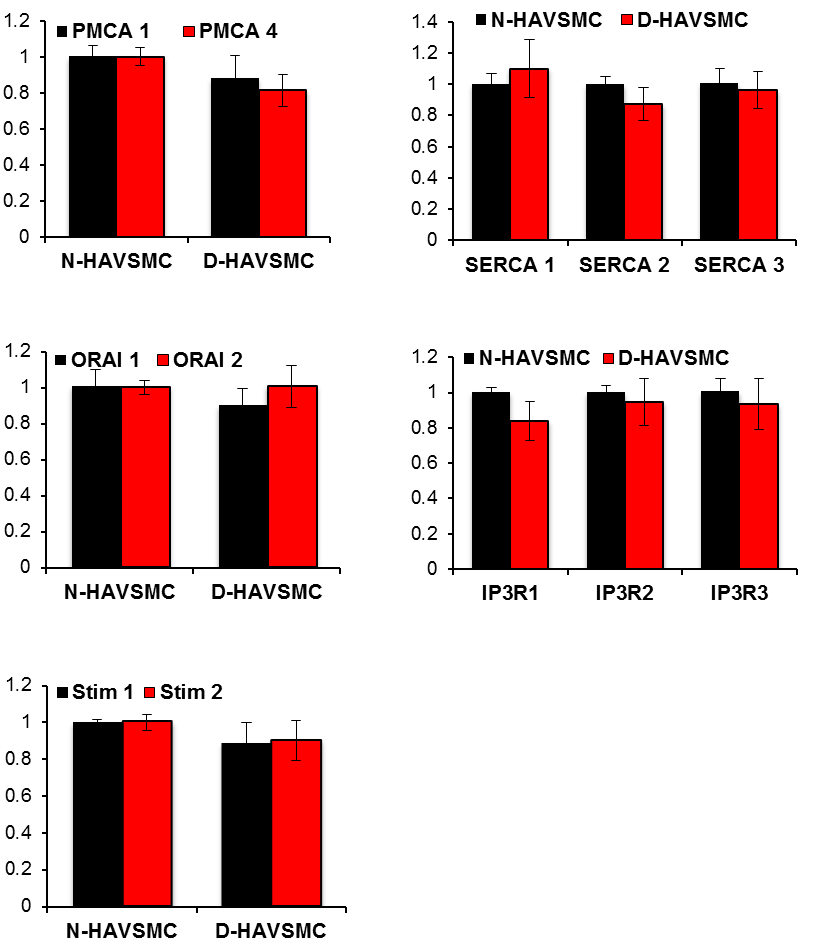
**
